# Functional Characterization of OXYL, A SghC1qDC LacNAc-specific Lectin from The Crinoid Feather Star *Anneissia Japonica*

**DOI:** 10.3390/md17020136

**Published:** 2019-02-25

**Authors:** Imtiaj Hasan, Marco Gerdol, Yuki Fujii, Yasuhiro Ozeki

**Affiliations:** 1 Graduate School of NanoBio Sciences, Yokohama City University, 22-2 Seto, Kanazawa-ku, Yokohama 236-0027, Japan; hasanimtiaj@yahoo.co.uk; 2 Department of Biochemistry and Molecular Biology, Faculty of Science, University of Rajshahi, Rajshahi 6205, Bangladesh; 3 Department of Life Sciences, University of Trieste, Via Licio Giorgieri 5, 34127 Trieste, Italy; mgerdol@units.it; 4 Graduate School of Pharmaceutical Sciences, Nagasaki International University, 2825-7 Huis Ten Bosch, Sasebo, Nagasaki 859-3298, Japan; yfujii@niu.ac.jp

**Keywords:** N-Acetyllactosamine (LacNAc), *Anneissia japonica*, anti-biofilm activity, cell adhesion, crinoid, Echinoderm, feather star, lectin, signal transduction, sghC1qDC

## Abstract

We identified a lectin (carbohydrate-binding protein) belonging to the complement 1q(C1q) family in the feather star *Anneissia japonica* (a crinoid pertaining to the phylum Echinodermata). The combination of Edman degradation and bioinformatics sequence analysis characterized the primary structure of this novel lectin, named OXYL, as a secreted 158 amino acid-long globular head (sgh)C1q domain containing (C1qDC) protein. Comparative genomics analyses revealed that OXYL pertains to a family of intronless genes found with several paralogous copies in different crinoid species. Immunohistochemistry assays identified the tissues surrounding coelomic cavities and the arms as the main sites of production of OXYL. Glycan array confirmed that this lectin could quantitatively bind to type-2 N-acetyllactosamine (LacNAc: Galβ1-4GlcNAc), but not to type-1 LacNAc (Galβ1-3GlcNAc). Although OXYL displayed agglutinating activity towards *Pseudomonas aeruginosa*, it had no effect on bacterial growth. On the other hand, it showed a significant anti-biofilm activity. We provide evidence that OXYL can adhere to the surface of human cancer cell lines BT-474, MCF-7, and T47D, with no cytotoxic effect. In BT-474 cells, OXYL led to a moderate activation of the p38 kinase in the MAPK signaling pathway, without affecting the activity of caspase-3. Bacterial agglutination, anti-biofilm activity, cell adhesion, and p38 activation were all suppressed by co-presence of LacNAc. This is the first report on a type-2 LacNAc-specific lectin characterized by a C1q structural fold.

## 1. Introduction

Molecular recognition is one of the most essential mechanisms used to regulate cell systems. In primitive organisms, in specific cell types and in early developmental stages, glycans (monosaccharide chains) located on the cell surface act as a fundamental sugar code for recognition. Indeed, specific glycan structures are initially recognized by glycans (via carbohydrate–carbohydrate interaction) [1] and, subsequently, by lectins (via carbohydrate–protein interaction). Virtually all living organisms are endowed with lectins (glycan-binding proteins) that enable such molecular interactions in different biological contexts.

Echinodermata are the second largest phylum in the Deuterostomia lineage and, due to their position in the metazoan tree of life, they can be considered as relatives of chordates and vertebrates. To date, a large number of lectins have been isolated from members of the subphylum Eleutherozoa, containing mobile echinoderms belonging to the classes Echinoidea (sea urchins), Asteroidea (sea stars), Holothuroidea (sea cucumbers), and Ophiuroidea (brittle stars). These structurally different molecules pertain to different protein families, such as galectin [2], C-type [3,4], SUEL/RBL-type [5,6], R-type [7], and HSP110-type [8] lectins. Numerous studies have reported that invertebrate lectins are able to inhibit or to promote mammalian cell growth [9,10,11,12]. Moreover, some lectins are capable of killing carcinoma cells and microorganisms through the binding to N-acetylhexosamines, such as N-acetyl D-galactosamine (GalNAc), N-acetyl D-glucosamine (GlcNAc), N-acetyl D-mannosamine (ManNAc) and N-acetylneuramic acid (NeuAc) which in turn activates signal transduction, leading to cell death. Lectins obtained from echinoderms can regulate cell growth in different ways. For example, echinoidin, a C-type lectin found in the coelomic fluid of sea urchins, exerts its cell adhesion activity via the tripeptide motif RGD, known as a cell adhesive signal [13]; on the other hand, the SUEL/RBL-type lectin found in sea urchin venom promotes mitogenesis [14] and an R-type lectin isolated in sea cucumber has hemolytic properties [7].

Compared with other echinoderms, the class Crinoidea (subphylum Pelmatozoa) has been almost completely neglected, as far as lectin research is concerned. The morphology of crinoids resembles that of flowering plants due to the presence of hundred feather-like pinnules attached to their crown of arms. Fossil record, as well as molecular phylogeny, indicate that crinoids are the most primitive type of existing echinoderms. The oldest crinoid representative had already emerged in the mid-early Ordovician period of the Paleozoic era (542–251 million years ago). After becoming nearly extinct at the end of the Permian period mass extinction, crinoids recovered in the early Triassic, in the Mesozoic era (251–66 million years ago), and have subsequently undergone diversification to the present level [15,16]. The two extant forms or crinoids, which include feather stars (free-swimming organisms) and sea lilies (sessile animals that possess a stalk to attach to rocks), are both regarded as living fossils. Despite their ancient evolutionary origins, crinoids have a complex nervous system and possess an excellent ability of tissue-regeneration, which is particularly evident in their arms [17]. Since these properties are potentially useful for biological and medicinal studies, large efforts have been endeavored to develop protocols that could ensure a large-scale annual supply of individuals as an experimental animal model [18]. The improved study of lectins in this class of echinoderms could enable to clarify some aspects of deuterostome evolution through an improved view on lectin-glycan interactions.

In a previous study, we reported the discovery of OXYL, a Ca^2+^-independent lectin isolated from the arms of the feather star *Oxycomanthus japonicus* (family Comatulidae), a species whose scientific name has been recently updated to *Anneissia japonica*. This family includes four subfamilies, 21 genera and approximately 95 species. Comatulidae is the most commonly encountered and species-rich crinoid family in coastal regions and tropical coral reefs, particularly in the Indo-Western Pacific region [19]. The isolated lectin was soluble and consisted of a 14 kDa polypeptide. Frontal affinity chromatography showed that this molecule recognized type-2 N-acetyllactosamine (LacNAc: Galβ1-4GlcNAc), a common structure of complex-type glycans found in vertebrate Asn(N)-type glycoproteins and glycosphingolipids [20]. However, the primary structure of the lectin, its tissue localization and effects in vertebrate cell proliferation were unknown at the time of its initial discovery.

We took advantage of the availability of genome and transcriptome sequence data from *A. japonica*, resulting from a study carried out by the Brown University in 2014 [21], to investigate in detail the primary structure of OXYL, which was revealed as a protein belonging to the sgh(secreted globular head) C1qDC (complement (C)1q-domain-containing) protein family. We also found that OXYL was mainly localized in the tissues surrounding the coelom (the main body cavity) and in the arms, which suggests a possible function as an innate immune molecule.

In this study, we further show that OXYL pertains to a lineage-specific subfamily of C1qDC proteins that specifically evolved in crinoids, but not in other Eleutherozoa. Despite its ability to recognize target glycans on the cell surface, OXYL did not to show antibacterial or cytotoxic effects. This suggests that the activation of metabolic pathways by the interaction between the lectin and its ligands did not lead to the activation of apoptotic pathways in the target cells. We discuss the evolutionary implications of the glycan-binding properties of OXYL in this basal deuterostome, keeping in mind the fundamental role C1q proteins carry out at the crossroads between acquired (antibody-based) and innate immunity in vertebrates.

## 2. Results

### 2.1. Structural Characterization of OXYL as a sghC1qDC Protein

**Figure 1:**
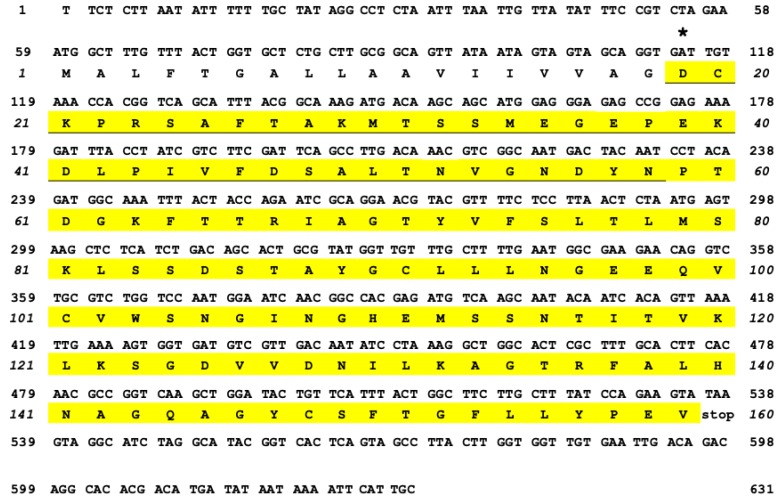
cDNA sequence and deduced amino acid sequence of OXYL. The asterisk (^19^Asp) indicates the first N-terminal amino acid of the mature lectin (yellow). The amino acid sequence identified by Edman degradation is underlined.

The N-terminal region of OXYL, including the first 40 amino acids of the mature protein, was determined by Edman degradation, with a repetitive yield of 87.59% (Figure S1). This partial amino acid sequence found a perfect hit with a virtually-translated 778-nucleotide long genomic contig, which contained a single 480 nucleotides-long open reading frame (Figure 1), with no introns. Unfortunately, the high fragmentation of the *A. japonica* genome assembly, which might be due to a combination of the high heterozygosity of echinoderms and the low sequencing coverage, prevented a reconstruction of the complete locus, covering the entire 5′ and 3′ untranslated regions (UTRs). At the present stage, the OXYL contig includes a likely incomplete 58 bp 5′UTR region and 240 bp downstream to the translation termination signal, which includes a potential polyadenylation signal in position 617–622, followed shortly thereafter by a CA sequence (position 631–632) and a T-rich region, which represent the canonical consensus for recognition by polyadenylation factors [22]. Altogether, these observations suggest that the 3′UTR of the OXYL mRNA is 93 bp long. The predicted sequence of the OXYL mRNA has been deposited in GenBank under the accession ID MK434202. No sequence corresponding to OXYL could be identified in the ovary transcriptome of *A. japonica*, indicating the lack of expression of this gene in this tissue.

The encoded protein consists of 159 amino acids and contains a N-terminal signal sequence (^1^Met-^18^Gly), which was inferred to undergo proteolytic cleavage through the secretory pathway, as the N-terminal residue of the mature polypeptide was determined to be ^19^Asp (Figure 1, asterisk). This is consistent with SignalP prediction, which identified a putative cleavage site with high confidence in the same position. The polypeptide displayed a candidate site for N-glycosylation (Asn-X-Thr), including the residues ^58^Asn-^60^Thr. However, the yield of ^58^Asn (126 pmol) quantitatively obtained by Edman degradation (Figure S1) indicates that this Asn residue did not undergo translational modification. The secreted OXYL polypeptide was therefore predicted to include, following single peptide cleavage, 141 amino acids, with a molecular mass of 15193.2.

**Figure 2:**
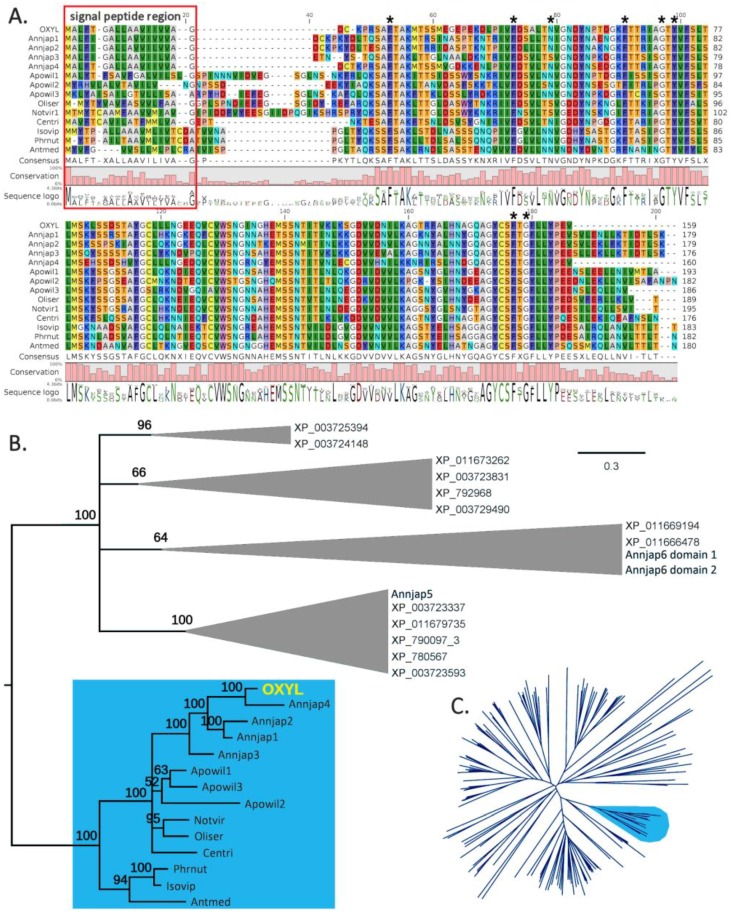
Panel **A**: Multiple sequence alignment of the full-length OXYL precursor and homologous sequences from other crinoid species. Histogram bars represent amino acid conservation in each position of the alignment. The signal peptide region is indicated in a red box. The 8 residues typically conserved in C1qDC proteins are marked with an asterisk. Panel **B**: Bayesian phylogenetic tree of OXYL-like sequences. Multiple sequences obtained from the same species are indicated with progressive numbers. The tree was rooted by using the C1qDC protein sequences from the genome of the sea urchin *Strongylocentrotus purpuratus* (indicated with GenBank accession codes, starting with “XP_”) and the three C1q domains from the two C1qDC proteins identified in the *A. japonica* transcriptome, but unrelated to OXYL. For simplicity’s sake, outgroup sequences have been collapsed in cartoons. Numbers close to each node represent posterior probabilities. The OXYL clade is indicated with a blue background. Panel **C**: neighbor joining tree of all C1qDC sequences from echinoderms, based on the multiple sequence alignment of the C1q domain (see materials and methods for details). The OXYL clade, supported by bootstrap value = 94, is indicated with a blue background. Each sequence is designated with a six letter code, indicating the first three letters of the genus and species name (see materials and methods for a complete list of sequences).

A C1q domain (Pfam family: *C1q* (PF00386)) could be recognized with high confidence by Hmmer (e-value = 5.9E^−22^), starting immediately after the signal peptide, and comprising all the remaining part of the polypeptide. The 8 residues (^26^Phe, ^46^Phe, ^52^Asn, ^64^Phe, ^70^Gly, ^72^Tyr, ^150^Phe, and ^152^Gly) that are typically conserved in all C1q proteins [23] were also present in OXYL (Figure 2A, asterisks). The precursor protein lacks both collagen-like regions (found in many vertebrate C1qDC proteins) and coiled-coiled regions (found in many invertebrate C1qDC proteins). Therefore, based on a previously suggested classification scheme for C1qDc proteins, OXYL should be categorized as a sgh(secreted globular head)C1q protein [24,25].

### 2.2. OXYL is Part of a Multigenic Family of sghC1qDC Proteins Restricted to Comatulida

Our investigation permitted to identify several additional genes encoding proteins sharing high similarity with OXYL in the *A. japonica* genome, even though the high fragmentation of this resource did not allow an exhaustive discrimination between paralogous gene copies and allelic variants, also preventing the full-length reconstruction of two gene sequences. In detail, the *A. japonica* genome contains four other complete genes closely related to OXYL, all predicted to be intronless. These genes encode precursors of similar size (159–180 amino acids), all classifiable as sghC1qDC proteins, sharing 58–87% sequence homology in pairwise comparisons (Figure 2A). Such a divergence, together with the origins of the genome data from a single individual, is consistent with the presence of multiple paralogous gene copies.

Previous studies have revealed that marine invertebrates can contain an extremely variable number of C1qDC genes, ranging from just a very few to several hundred [25], as this family underwent multiple lineage-specific expansion events along its evolution. The screening of echinoderm genomes indicated that these animals possess a moderate number of C1qDC genes, compared with other metazoans. Namely, we could detect 6 genes in the sea cucumber *Apostichopus japonicus*, 14 genes in the sea urchin *Strongylocentrotus purpuratus*, and a much larger number (59) in the sea star *Acanthaster planci*. Although the number of C1qDC sequences found in a transcriptome most certainly depends on multiple factors, e.g., tissue of origin, sequencing depth, and others, the data collected from crinoid transcriptomes can provide a rough estimate of the number of sghC1qDC genes present in this class of echinoderms. This number varied considerably from species to species, ranging from 2 (in the *A. japonica* ovary transcriptome) to 50 (in the *Notocrinus virilis* arm transcriptome). While the domain architecture of the encoded proteins varied to some extent, we could detect sequences orthologous to OXYL in seven other species pertaining to the order Comatulida, namely *Antedon mediterranea*, *Aporometra wilsoni*, *Cenolia trichoptera*, *Isometra vivipara*, *Notocrinus virilis*, *Oligometra serripinna,* and *Phrixometra nutrix* (Figure 2A). With the exception of *A. wilsoni*, where three paralogous sequences were found, the transcriptomes of all the other species only possessed a single OXYL-like sequence. All OXYL-like sequences share, at the amino acid level, pairwise sequence identity higher than 40% and possess very similar length and domain organization. On the other hand, no expressed sequence closely related to OXYL was found in members of the orders Cyrtocrinida, Hyocrinida and Isocrinida.

Sequence clustering approaches placed all Comatulida OXYL-like sequences in a well-supported clade (bootstrap support = 94, indicated in light blue in the radial tree in Figure 2C). This clade did not include any C1qDC sequence neither from other crinoids, nor from Echinozoa and Asterozoa. Bayesian inference analysis (Figure 2B): (i) confirmed the lack of closely related sequences in other non-crinoid echinoderms (for simplicity’s sake, only sea urchin sequences have been included in this tree); (ii) identified a small subgroup of sequences (Isovip, Phrnut and Antmed in Figure 2B) with peculiar features, all pertaining to species in the Antedonoidea superfamily; (iii) strongly supported the monophyly of the five full-length OXYL-like genes identified in *A. Japonica*.

### 2.3. Tetrameric Structure

**Figure 3:**
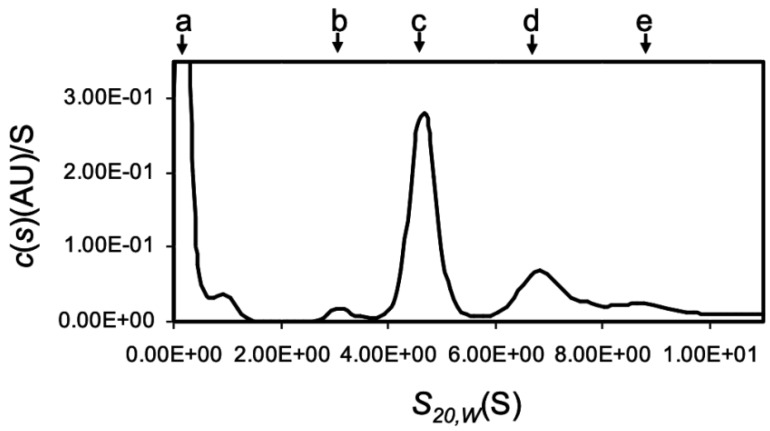
Multimeric structure of OXYL by using analytical ultracetrifugation. Distribution of sedimentation coefficient c(s20,w) by sedimentation velocity AUC. Calculated c(s) was plotted versus s20,w the sedimentation coefficients corrected to 20°C in water. **a**: very likely come from salt and/or buffer, **b**: 3.28 S (37 kDa, dimer), **c**: 4.88 S (65.8 kDa, tetramer), **d**: 7.25 S (121 kDa, octemer), and **e**: 9.19 S (178 kDa, dodecamer). The experiment was performed with 0.4 mg/mL protein.

The analytical ultracentrifugation showed that OXYL was mainly found in a tetrameric form (Figure 3), although sghC1qDC family proteins are generally known to associate as trimers. Among the peaks of c(s), the large peak observed near 0 S was most likely originated from the gradient of buffer solution and/or the high concentration salt (Figure 3a). The main peak of c(s) was evident at 4.88 S (Figure 3c, 65.8 kDa: 70.3%). In addition, minor peaks of c(s) were also present at 3.28 S (Figure 3b, 37 kDa: 13.5%), 7.25 S (Figure 3d, 121 kDa: 27%), and 9.16 S (Figure 3e, 178 kDa: 13.5%), respectively (Figure S2). Besides the main tetrameric form, these results highlighted that the tetrameric OXYL was further associated to form octamer and dodecamer, in addition to a minor fraction of lectin associated as a dimer. The finding that OXYL is predominantly found as a tetramer is further supported by observations previously reported based on gel permeation chromatography [20]. Furthermore, this analysis suggested that OXYL is in dynamic equilibrium between tetramer and octamer. The frictional ratio (f/f_0_) of OXYL is equal to 1.23, indicating that the protein is globular supporting the organization of OXYL as a sghC1qDC protein.

### 2.4. Generation of Antiserum Against OXYL

**Figure 4:**
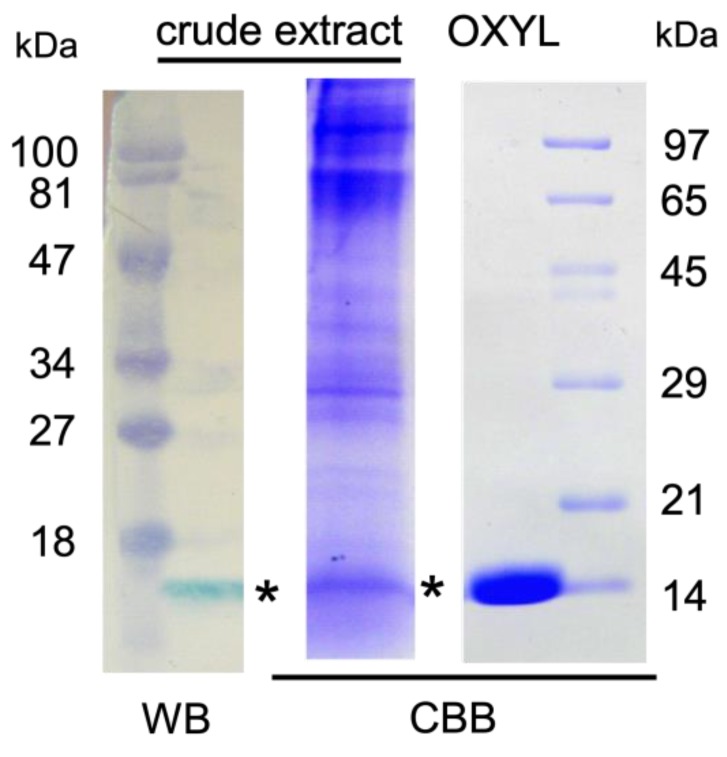
Detection of OXYL by antiserum. *A. japonica* arm extract (crude extract) and purified lectin (OXYL) were separated by SDS-PAGE and transferred to a membrane. OXYL (asterisk) was detected by peroxidase staining of HRP-conjugated goat anti-rabbit IgG raised against OXYL and by Coomassie Brilliant Blue R-250. Numbers at left (pre-stained) and right: molecular mass (×1000) standard.

Anti-OXLY antiserum obtained from immunized rabbits was used to identify OXYL by western blotting. Proteins obtained from the crude extract from the arms of *A. japonica* were separated by SDS-PAGE and blotted on a PVDF membrane. A band with a molecular mass of 14,000, detected by HRP-conjugated goat anti-rabbit IgG (Figure 4, crude extract), was evident at the same molecular weight of purified OXYL, stained with Coomassie brilliant blue (Figure 4, OXYL). The obtained antiserum allowed to identify the arms as the main site of localization of OXYL in the feather star.

### 2.5. Tissue Localization of OXYL

**Figure 5:**
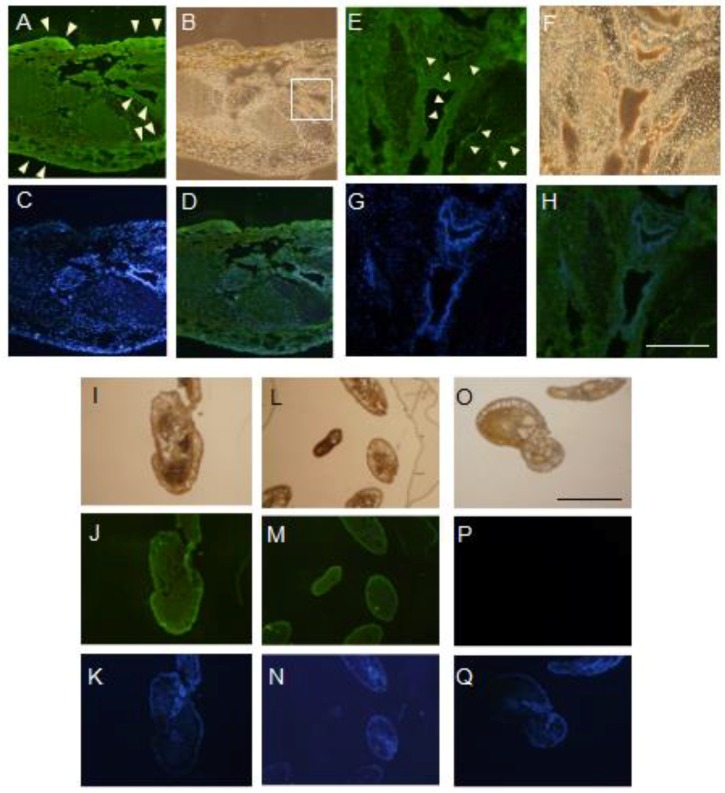
Localization of OXYL in *A. japonica*. Paraffin-embedded serial sections were stained by hematoxylin-eosin and anti-OXYL rabbit antiserum (**A**–**N**) and pre-immune rabbit serum (**O**–**Q**) followed by FITC-conjugated secondary anti-rabbit IgG goat antibody, and observed by fluorescence microscopy. Detections: FITC (**A**,**D**,**E**,**H**,**J**,**M,**,**P**), DAPI (**C**,**D**,**G**,**H**,**K**,**N,**,**Q**) and hematoxylin-eosin staining (**B**,**F**,**I**,**L**,**O**). The square in panel B was zoomed-in E-H. Scale bars: 100 μm (white) and 300 μm (black), respectively.

In this study, the arm tissue was decalcified during the preparation of tissue sections from adult feather stars, since the raw tissue displayed a remarkable hardness. Even though such procedure could have produced some artifacts, the expression of OXYL appeared to display a remarkable pattern of distribution. The detection of OXYL signal by the antiserum indicated its presence in the regions surrounding the coelom (Figure 5A,E) and in spicules (Figure 5G,M). These signals were overlapping with the DAPI signal (Figure 5D,H,K,N), indicating that the lectin was produced by proliferating cells. Since OXYL is a secretory protein (Figure 1) that shows high solubility (Figure 4), its presence in such tissues can be explained even in absence of its glycan ligands. The SUEL/RBL-type D-Gal-binding lectin from the sea urchin *Heliocidaris crassispina*, which is expressed in unfertilized eggs and secreted in the extracellular matrix, is known to play a key regulatory role in early embryonal development, from the fertilization to the gastrulation stage [26]. A different D-Gal-binding lectin (echinonectin), isolated in another sea urchin species (*Lytechinus variegatus*) and displaying Del-1/lactadherin-like (discoidin-like) primary structure, was also found to be expressed in the early embryo and secreted into the extracellular matrix. Its ability to adhere to cells suggested a possible function in the control of cell movement [27]. In contrast to these lectins, which are exclusively expressed in early developmental phases, OXYL was also expressed in adult tissues. This observation supports the involvement of lectins pertaining to different structural families in specific roles, which may be covered in different life stages and different tissues in the phylum Echinodermata.

### 2.6. OXYL Quantitatively Binds to Type-2 N-LacNAc

**Figure 6:**
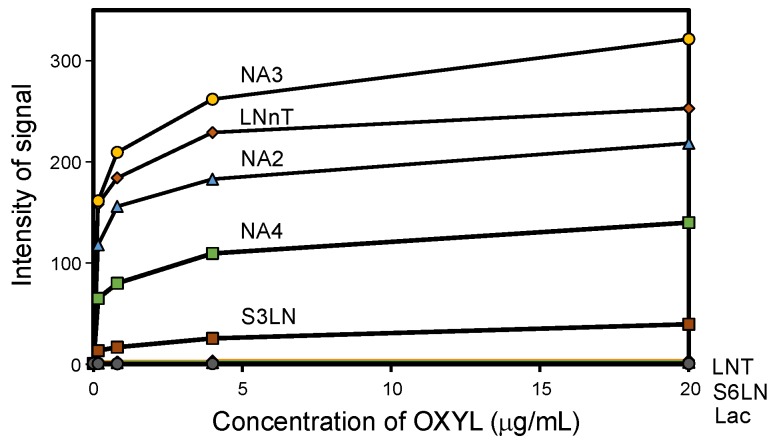
Glycan-binding properties of OXYL. Fluorescence-labeled OXYL was subjected to glycan array analysis using glycochip, where 8 glycan structures were immobilized. Fluorescence signals for the 8 glycans (listed in Table 1) are represented as signal intensities.

Glycan array analysis, carried out to investigate the binding specificity of OXYL, led to the same results previously obtained by frontal affinity chromatography [20]. The glycans analyzed in the microarrays in this study are listed in Table 1 (see also Figure S3). OXYL was shown to bind to N-neo tetraose (LNnT: Galβ1-4GlcNAcβ1-3Galβ1-4Glc), bi- (NA2), tri- (NA3) and tetra-antennary (NA4) N-glycans (Figure 6). All these sugars possess type-2 LacNAc (Galβ1-4GlcNAc) (Figure 6). On the other hand, OXYL did not bind neither to lacto N-tetraose (LNT: Galβ1-3GlcNAcβ1-3Galβ1-4Glc), with type-1 LacNAc (Galβ1-3GlcNAc), nor to lactose (Galβ1-4Glc), without an N-acetyl group (Figure 6, LNT). OXYL could bind to 3-sialylated LacNAc more strongly than to 6-sialylated LacNAc (Figure 6, S3LN versus S6LN). However, since this interaction was much weaker than that observed with type-2 LacNAc (Figure 6, S3LN versus LNnT), it can be hypothesized that the free C-6 hydroxyl group of Gal in type-2 LacNAc is essential for OXYL binding. The specific binding of OXLY to type-2 LacNAc was further confirmed by hemagglutination inhibition assays (Table 2, Galβ1-4GlcNAc versus Galβ1-4Glc). Hemagglutination was not inhibited by monosaccharides (Gal and NeuAc), β-galactoside disaccharides without an N-acetyl group (lactose). In addition, neither the addition of glycans, such as chondroitin sulphate and heparine, nor the presence of *P. aeruginosa* lipopolysaccharide or *M. luteus* peptidoglycan interfered with hemagglutionation (Table 2).

### 2.7. OXYL Displays Bacteria Agglutination Properties

**Figure 7:**
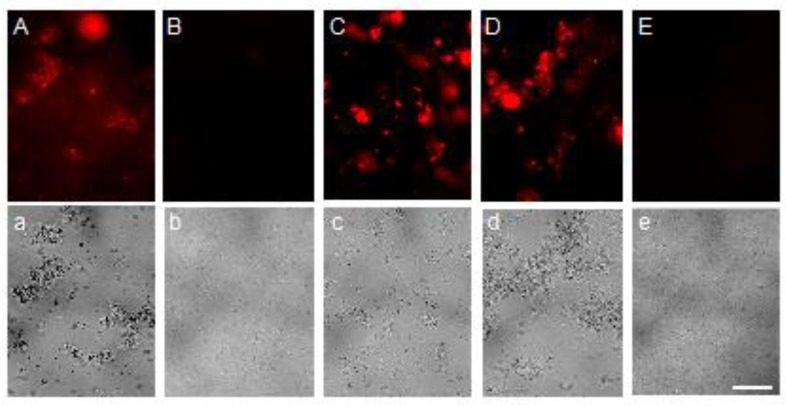
Agglutination of *Pseudomonas aeruginosa* by OXYL. Two micrograms of OXYL were administrated to the bacteria (**A**–**D**,**a**–**d**), which were observed by fluorescence microscopy (**A**–**E**; λ_ex/em_ = 550/566 nm) and phase-contrast microscopy (**a**–**e**). Ten mM LacNAc (**B**,**b**), lactose (**C**,**c**) or 0.5 mg/mL lipopolysaccharide (**D**,**d**) were added in co-presence with OXYL. E and e are negative controls without OXYL. The bar indicates 100 μm.

An assay carried out with HiLyte-555 fluoro-labeled OXYL showed that this lectin led to the strong agglutination of *P. aeruginosa* bacterial cells (Figure 7). This agglutination was specifically inhibited by the co-presence of LacNAc, which, as previously demonstrated, is one of the saccharides recognized by OXYL (Figure 7B,b). This specificity was confirmed by the lack of any inhibitory effect on agglutination in the co-presence of lactose (Figure 7C,c). At the same time, bacterial lipopolysaccharide did not inhibit the agglutination of *P. aeruginosa* (Figure 7D,d).

### 2.8. Antibiofilm Activity and Influence on Bacterial Growth of OXYL

**Figure 8:**
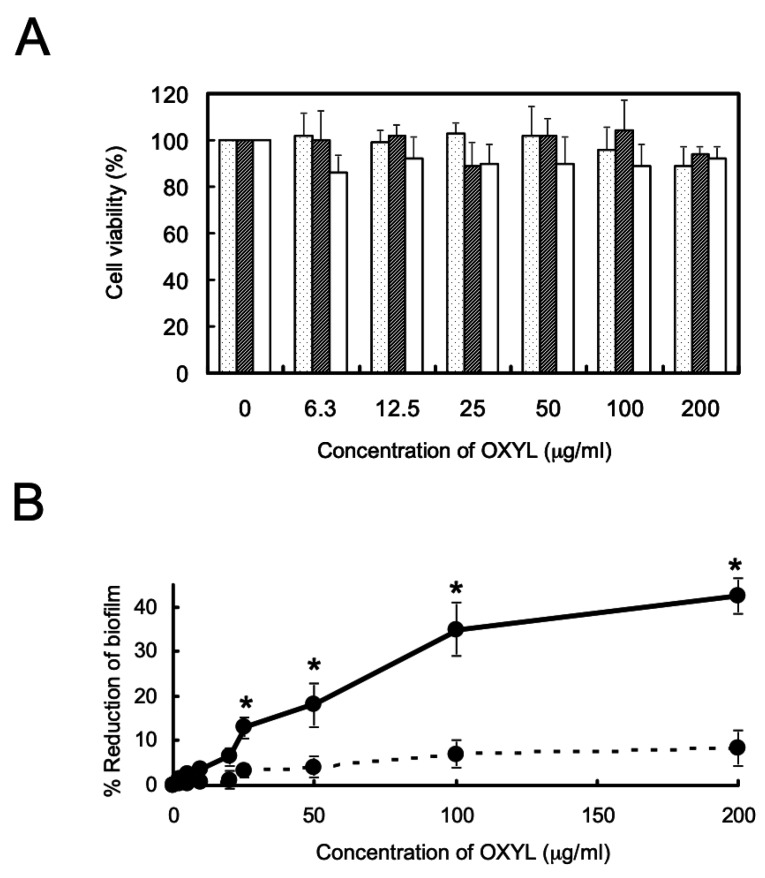
Influence of OXYL on bacterial growth, and its anti-biofilm activity. **A:** Bacteriostatic activity. Dotted, hatched, and white columns indicate the growth of *L. monocytogenes*, *S. boydii* and *P. aeruginosa*, respectively. Error bars: SE from three independent experiments (replicates). The data reported here are the mean ± SE (*n* = 3). **B:** Anti-biofilm activity. *P. aeruginosa* was cultured for 24 h with various OXYL concentrations (0–200 μg/mL) in 96-well plates in the absence (solid line) or presence (dotted line) of LacNAc (10 mM). * *p* < 0.05.

The presence of OXYL inhibited biofilm formation in *P. aeruginosa* (Figure 8A, solid line), even though it did not affect bacterial growth. As in the case of bacterial agglutination, anti-biofilm activity was inhibited by the co-presence of LacNAc (Figure 8B, black bar). We have previously reported a similar reduction of *P. aeruginosa* biofilm formation by another N-acetylhexosamine-binding lectin extracted from a sponge (*Halichondria okadai*) [12]. 

### 2.9. OXYL Adhered to Cells by Binding to LacNAc on the Cell Surface

**Figure 9:**
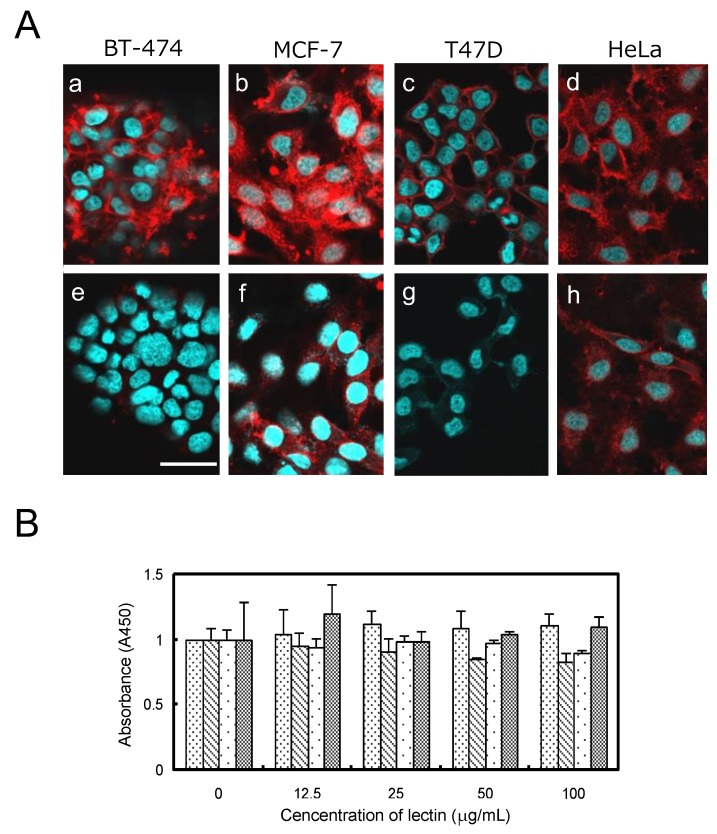
Binding and growth effect of OXYL to cancer cells. **A:** HiLyte Fluoro 555-conjugated OXYL (10 μg per cells) was administrated to BT-474 (**a**,**e**), MCF-7 (**b**,**f**), T47D (**c**,**g**), and HeLa (**d**,**h**) without (**a**–**d**) or with (**e**–**h**) LacNAc (10 mM). **B:** Cell viability of OXYL against four types of cancer cells. Cells were treated with OXYL at various concentrations (0–100 μg/mL) for 48 h, and viability was determined by WST-8 assay. Values for BT-474, MCF-7, T47D, and HeLa are shown by fine dotted, hatched, rough dotted and mesh bars, respectively.

HiLyte Fluoro 555-labeled OXYL could bind to BT-474 (breast), MCF-7 (breast), and T47D (breast) and HeLa (cervical), cancer cells (Figure 9Aa–d) cell lines. The fluorescent signal clearly outlined the contour shape of each cell, indicating that the lectin was bound to the cell surface via glycans containing LacNAc structures, which are commonly present on the membranes of mammalian cells. In agreement with the results reported in the previous section, OXYL binding was inhibited by the addition of LacNAc (Figure 9Ae–h). The amount and type of glycans on cell surface greatly differ depending on the cell line. MCF-7 and HeLa, the two cell lines where OXYL binding could not be completely inhibited by the addition of LacNAc, may be rich in branched N-type sugar chains and/or lacto-neo-tetraose glyco sphingolipid. Alternatively, these cells may be rich in NeuAcα2-3-linked glycans. Moreover, the fluorescence signal remained on the cell surface even when incubation lasted for a relatively long period of time (2 to 12 h). This behavior was clearly different from that observed for other N-acetylhexosamine-binding lectins, such as iNoL [10] and HOL-18 [12], which migrated inside the cells over time, triggering apoptosis. This observation is consistent with the complete lack of cytotoxicity of OXYL on bound cells (Figure 9B). These results provide useful evidence about the cellular function exerted by this lectin in crinoids, pointing out that cell binding is not necessarily associated with cytotoxicity.

### 2.10. OXYL LacNAc-Dependently Activated the p38 of BT-474 Cancer Cells 

**Figure 10:**
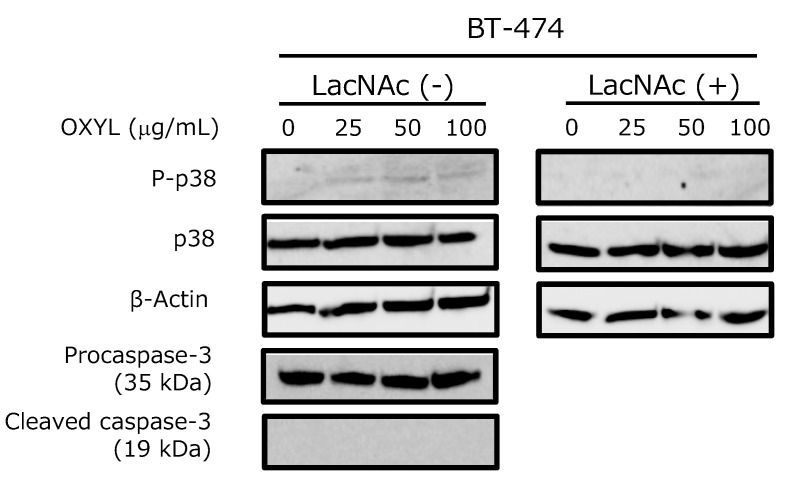
Effects of OXYL on phosphorylation and expression levels of p38 and caspase-3 in the BT-474 cell line. Cells (4 × 10^5^ in each experiment) were treated with various concentrations of OXYL (0–100 µg/mL), and activation levels were evaluated by western blotting of lysates in absence (−) and presence (+) of LacNAc (10 mM). Experiments were performed in triplicates.

The activation of p38, one of the most important kinases in the MAPK pathway, was moderately induced by OXYL in BT-474 cells in a dose-dependent manner (Figure 10, P-p38 versus p38). This behavior may be explained by the involvement of p38 in a secondary signaling pathway, only indirectly activated by OXYL binding, which would be consistent with the relatively weak degree of phosphorylation observed in this study. This effect was cancelled by the co-presence of LacNAc, which has been demonstrated as the ligand of OXYL by western blotting (Figure 10, LacNAc (−) versus LacNAc (+)). Nevertheless, OXYL did not induce the activation of caspase-3 (Figure 10, Pro-caspase-3 versus Cleaved caspase-3). Moderate levels of phosphorylated p38 may not have been sufficient to trigger activation of caspase-3 and apoptosis. A number of animal lectins are able to induce signal transduction, with the activation of both MAPK pathway and caspases [10,12,28]. On the other hand, ability of some lectins to activate the MAPK pathway without affecting caspases has been also reported in literature. For example, Gb3-binding lectins do not necessarily produce cytotoxic effects against Gb3-expressing cells. Indeed, SAL (*Silurus asotus* lectin), a lectin pertaining to the SUEL/RBL family, isolated from catfish eggs, activated MAP kinases in Burkitt’s lymphoma cells through Gb3 binding, but did not trigger apoptosis [29]. OXYL proved to be another example of a lectin that binds to specific glycans exposed on cell surface, activating the metabolic system without induction of cell death.

## 3. Discussion

We identified secreted globular head C1q (SghC1q) protein as a major lectin family in the crinoid class (Figure 1). This result is in contrast with the previous observation that C-type lectins, such as “echinoidin”, represent the main glycan-recognition molecules in the coelomic fluid of species from the subphylum Eleutherozoa [3,4]. This suggests that a certain degree of flexibility exists in echinoderms about the structural architecture of secretory lectins that may cover important functions, such as immune recognition. The primary structure obtained from Edman degradation and bioinformatics analysis of genome data highlighted several interesting points of contact and divergence with other C1q domain-containing (C1qDC) proteins previously identified in other invertebrates. Indeed, immunostaining indicated a certain degree of tissue-specificity and the fact that OXYL mRNA was not expressed in the ovary tissue confirms that the lectin is produced only in specific tissues. Furthermore, unlike most bivalve C1qDC genes [25], the crinoid gene encoding OXYL was intronless, as no intron was found in the region separating the signal sequence from the globular C1q domain. Whether this is a general feature of all crinoid C1qDC genes, or it is a specific characteristic of OXYL will be clarified by further bioinformatics analyses, once a complete crinoid genome will become available.

Our study indicated the presence of at least four other paralogous OXYL gene copies in this species, as well as several orthologous sequences in other species of the order Comatulida, suggesting that this molecule plays an important role in crinoids (Figure 2). Future studies should be directed at clarifying the functional meaning of this gene family expansion event, aiming to investigate whether any of these genes is transcriptionally responsive to environmental changes, such as infection and change of water quality. C1qDC genes are well known to be responsive to immune challenges and stress conditions in bivalves, where they are part of largely expanded gene families [30,31,32], but additional evidence has linked them to the complex inter-specific interactions in other invertebrates, such as the response to predator kairomones in marine snails [33]. The echinoderm immune defense system is one of the best characterized in invertebrates, as multiple key components of immune recognition, signal transduction and antimicrobial effectors have been identified over the years [34,35]. This wealth of information may enable to chase down the possible function of OXYL and its interaction with other immune receptors and effectors.

The C1q domain is found in a large number of metazoan proteins, such as the main chains of the C1q complex, collagens, adiponectins, hibernation-specific protein, regulator of synapse organizer and also several lectins [36,37]. This domain is characterized by a jelly-roll fold of β-sandwich structure consisting of 10 β-strands. This topology is also found in proteins pertaining to the TNF superfamily, even though the primary structures of TNF and C1q do not share any significant similarity [38], in addition to galectins and legume lectins [39], and TNF-like bacterial lectins [40]. It seems likely that this fold may be used as a common structural scaffold with high potential for intermolecular interaction. Although the origins of the C1q domain are still unclear, this fold is found in both protostome and deuterostome animals, and it is also present in some bacteria. Since C1qDC lectins have been isolated in different animals, from the comparative glycobiology viewpoint metazoans have used C1q as a recurrent tool for glycan-binding. In contrast with most C1qDC lectins described in literature, which require divalent cations for their activity, OXYL did not require them at all for its carbohydrate-binding activity.

The sghC1qDC architecture, which characterizes proteins with an N-terminal signal sequence for secretion and without a collagen tail, have been reported in a number of vertebrate and invertebrate animals, and in some cases they work as lectins [24]. In mollusks, such proteins have been isolated as lectins binding to NeuAc, D-Man and lipopolysaccharide [41,42,43]. In chordates, D-Fuc [44] and D-GlcNAc [45] binding lectins have been purified from Petromyzontiformes and Osteichthyes, respectively. However, several studies have recently started to also unveil the glycan-binding properties of human C1qDC proteins. For example, the galactosylation of complex-type N-linked glycans in the Fc region of human IgG has been demonstrated to improve the binding of this immunoglobulin with C1q [46]. Moreover, C1qDC proteins are known to bind to monosaccharides [47] and glycosaminoglycan [48], and the human adiponectin can bind to bacterial lipopolysaccharides [49]. Considering the weight of such evidence, the ancestor of C1q was likely to be a carbohydrate-binding protein, which may have acquired other characteristic features along with evolution in different lineages.

Glycan array assays and the inhibition test demonstrated that OXYL specifically recognized type-2 LacNAc (Figure 6, Table 1 and Table 2). On the other hand, the lectin did not bind to monosaccharide or lactose, proving further evidence in support of its specific recognition of the N-acetyl group in GlcNAc. These recognition properties are clearly different from those of RCA 120, ECA and galectin, which are all known as LacNAc-binding lectin being also able to bind to lactose. This result resembles the observations made for echinoidin, which can bind to the core structure of the mucin-type glycan, without binding to the monosaccharide [3]. The diverse carbohydrate-binding properties of lectins derived from marine invertebrates, might be exploited in the future with the aim to discover new drugs or to develop new diagnostic tools for cellular biology.

Another peculiar feature of OXYL was the finding that it adopts tetrameric structure, as revealed by ultracentrifugation, unlike most C1qDC proteins, which are mostly organized as trimers [34]. This uncommon result found strong experimental confirmation in the results obtained by the gel permeation chromatography results reported in a previous study [20]. In addition to the tetrameric form (Figure 3c), OXYL seemed to be able to reversibly adopt other associative forms, from dimers to dodecamers (Figure 3b,d,e). The ability to create homo- or heterotrimeric assemblies is a well-known property of many C1qDC proteins, including the three main chains of the human C1q complex, cerebellin, and adiponectin [50,51,52]. Such proteins can also create higher-order hexametric complexes, whose formation is usually mediated by intermolecular disulfide bonds. The associative state of the C1q subunits has a strong impact on the biological properties of such complexes. For example, adiponectin is able to stimulate the activity of AMPK only in its trimeric form, whereas the hexameric form is the only one capable of triggering NF-kB signaling [53,54]. The assembly of higher-order “bouquet of tulips” super-structures, mediated by N-terminal collagen regions, is also of primary importance to enable the activation of the classical pathway of the complement system by the C1q complex [55]. In light of these observations, the presence of multiple associative forms of OXYL is of great interest, as the predominant tetrameric form may exert different biological activities compared to the less abundant dimeric, octameric, or dodecameric OXYL. Moreover, it is presently unknown whether heteromeric complexes between OXYL and its paralogous proteins (Figure 2A) can be created, or such assemblies are strictly homomeric. Altogether, the complex and still poorly characterized associative properties of OXYL may translate into a fine regulation of immune response upon pathogen recognition in this species.

Although several sghC1qDC proteins have been so far identified in invertebrates, very few studies have investigated the status of association of their subunits. There is little doubt that the accumulation of physicochemical knowledge on the structural properties of C1qDC proteins with carbohydrate-binding activity, as well as the elucidation of the structural features of their multimeric assemblies will improve our understanding of the biological role of these molecules.

Immunohistochemistry showed that the expression of OXYL was mainly detectable around the coelomic cavities and in the peripheral areas of skin in the arms. This suggests that this lectin exerts its biological functions in the outer and humoral environments, implying a possible involvement in body defense (Figure 5). Although only limited information is presently available concerning the localization of the different lectins isolated so far in the phylum Echinodermata [26,27], different types (C1q, SUEL/RBL, discoidin-like and C-type) of lectins display a variegated tissue distribution and pattern of expression during development. The accumulation of such information in the years to come will most likely help to better understand the evolutionary processes that have led to the functional diversification of invertebrate lectins compared with those found in vertebrates. The localization of OXYL in the close proximity of the coeloms and stromal tissues in the arms of the feather star is reminiscent of that of the mammalian C1q, which is expressed in the stroma and vascular endothelium of several human malignant tumors [56].

Although the C1q domain is mostly known as the characterizing domain of the C1q chains, primary mediators of immune response, which connect antigen-complexed IgGs with other complement factors in human, some studies have reported complement-independent antibacterial [57,58] or cancer growth promoting [59] activities by C1qDC proteins purified from fish and human, respectively. Although OXYL showed no direct antibacterial activity (Figure 8A), it strongly agglutinated *P. aeruginosa* cells (Figure 7A). Lipopolysaccharides are typical cell wall components and well-known virulence factors in Gram-negative bacteria [60], which may be recognized by some of C1qDC proteins acting as pattern recognition receptors [41,42,43]. However, the results of the assays carried out in this study demonstrate that the agglutination of *P. aeruginosa* by OXYL was LPS-independent, as this activity could be only inhibited by LacNAc (Figure 7B,D). The identification of the ligands specifically recognized on the bacterial cell surface by OXYL might help to understand the functional importance of the interaction between LacNAc-binding lectins and bacterial glycans in metazoan immune system.

OXYL, on the other hand, showed a remarkable anti-biofilm activity (Figure 8B). Similar properties have been previously observed in galactose-binding lectins isolated from snake venom and sea hare eggs [61,62], as both displayed anti-biofilm, but not antibacterial activity. Even though OXYL is not able to kill bacterial cells by itself, we hypothesize that its role in the context of crinoid immunity might be to facilitate the recognition of agglutinated bacteria by opsonins, enabling the elimination of the invading cells and allowing the intervention of circulating phagocytes thanks to the disruption of biofilm structure.

It is known that lectins found in plants, invertebrates and fishes, generally associated in dimeric or higher-order multimeric complexes, can influence the proliferation of mammalian cells. For example MytiLec-1, a dimeric lectin isolated from the mantle of mussels, can bind to human lymphoma cells, induced apoptosis. However, when the same lectin was dissociated in its monomeric state, it lost its agglutination and apoptosis-inducing properties [63]. Although OXYL displays a similar multimeric structure (Figure 3) and also shows a significant cell adhesion activity (Table 2, Figure 9A), it has no effect on cell growth (Figure 9B). SAL, a trimeric lectin isolated from catfish eggs, is another example of a lectin which can modulate cellular pathways upon the interaction with target cells. This lectin can trigger the activation of the intracellular MAP kinase system in Burkitt’s lymphoma cells, leading to a decreased expression of members of the ATP-binding cassette family, even though it cannot prevent cell proliferation [64]. The combined observation that (i) OXYL possess can activate MAP kinases, without affecting the activity of caspases (Figure 9), and that (ii) its cell-recognition properties depend on the binding of glycans having LacNAc structure, with no effect on cell proliferation (Figure 8), can be interpreted as follows. We hypothesize that the interaction between OXYL and the LacNAc glycans located on the surface of its target cells may trigger the activation of intracellular metabolism. This interaction probably activates presently unknown pathways unrelated to apoptosis though it is still linked with a moderate activation of the p38 kinase.

A comparative overview on the properties of OXYL and members of the galectin family might be important for understanding the role of this lectin. While some galectins can have a cytotoxic effect on the cells they recognize [65], OXYL did not suppress the growth of cancer cells. On the other hand, OXYL and galectins share a jelly roll topology, in spite of a complete lack of primary structure similarity. Galectins basically display binding properties to type-2 LacNAc. They selectively also recognize NeuAcα2-3LacNAc but not NeuAcα2-6LacNAc [66]. These properties reveal a glycan-binding specificity similar to that of OXYL (Figure 6). Functional studies have revealed that galectins interact with the hydroxyl group of the galactose found in C-6 position in lactose and did not interact with the hydroxyl group at the C-3 position [67]. In a similar fashion to galectins, OXYL may also interact with the hydroxyl group at the C-6 position, as explained by its stronger binding to LNnT, compared to S3LN (Figure 6). The detailed bonding structure will be clarified by the elucidation of the 3D structure of this molecule. After all, these two types of lectins share similar LacNAc-binding properties and the same stereoscopic topology.

In detail, C1q/TNF-family has been shown to bind to various glycans [46,47,48], to modify activation of MAP kinases, such as ERK and p38 [68,69], and to regulate the proliferation of cells. Although OXYL could not regulate cell growth by itself, it led to a moderate activation of p38 (Figure 10), maybe as a part of a secondary signaling pathway. The study of the immune system of lamprey, a key organism in the evolution of vertebrates, has brought new impulse to the study of C1q in animal immunity. This primitive jawless fish was found to produce a characteristic C1q protein (LC1q), which was discovered as a LacNAc-binding lectin during the search for the ancestral C1q molecule of chordates. LC1q was elucidated to specifically activate the complement system, activating cell lytic pathways in a different way compared with mammals [70]. In light of these observations, it can be suggested that the peculiar lamprey complement system evolved independently from that of jawed vertebrates, using a proto-typical C1q protein that acquired both immune and glycan-binding activities [45]. Although several sialic acid (NeuAc)-binding lectins described in invertebrate animals share a C1q-like fold [41], recent studies have revealed that the same binding function can be also achieved by lectins (SigLec) with an immunoglobulin-like fold [71]. The glycan-binding properties of lectins have long been considered to be strictly dependent on their unique primary structures. However, these results, together with the elucidation of the structural properties of C1q-like and immunoglobulin-like lectins, provide a new framework of conceiving the lectin structure/function relationship. Indeed, the widespread nature of these recurrent structural folds suggests an ancient origin in biological systems as an example of molecular bricolage [72]. Thanks to their remarkable binding properties, these structures may have been recruited as lectins, components of the complement system or recognition molecules of the adaptive immune system along evolution, based on the increasing need to recognize specific ligands with the colonization of new environments.

Although OXYL has been first isolated by basic biochemical methods in a previous study [20], we here provide the characterization of its structure as a member of the C1qDC family as a type-2 LacNAc-binding lectin. The comparative view among OXYL, LC1q, and other C1qDC proteins isolated in various invertebrates may lead to new relevant findings concerning the evolution of innate immunity in Deuterostomia. Moreover, in light of the emerging role of human C1q as cancer proliferation regulating factor, OXYL derivatives may also be also studied for the development of novel drugs in the field of oncology.

## 4. Experimental Design

### 4.1. Materials

*A. japonica* was supplied from Mrs Hisanori Kohtsuka and Mamoru Sekifuji in the Misaki Marine Biological Station, The University of Tokyo, Miura city, Sagami Bay, Kanagawa Prefecture, Japan, and stored at −80 °C until the beginning of this study. Strains of three bacteria (*Pseudomonas aeruginosa*, *Shigella boydii* and *Listeria monocytogenes*) were obtained from the Dept. of Biochemistry and Molecular Biology, University of Rajshahi, Bangladesh. Human cell lines BT-474 (breast cancer), MCF-7 (breast cancer), T47D (breast cancer), and HeLa (cervical cancer) were obtained from the American Type Culture Collection (Manassas, VA, USA). Lipopolysaccharide (from *P. aeruginosa*), peptidoglycan (from *Micrococcus luteus*), bovine serum albumin (BSA), Pathoprep-568, cell lysis buffer M, Mayer’s hematoxylin solution, eosin alcohol solution, 4% paraformaldehyde phosphate buffer, 20% glutaraldehyde solution, cacodylate buffer, Canada balsam, crystal violet solution, Penicillin-Streptomycin solution, and horseradish peroxidase (HRP)-conjugated β-actin mAb were all acquired from FUJIFILM Wako Pure Chemical Corp. (Osaka, Japan). Standard protein markers for SDS-PAGE were purchased from Takara Bio Inc. (Kyoto, Japan). HRP-conjugated goat anti-rabbit IgG was obtained from Tokyo Chemical Industry Co. (Tokyo, Japan), Type-2 LacNAc was obtained from Dextra Laboratories Ltd. (Reading, England). Prestained protein marker was from Bio-Rad Laboratories (Hercules, CA, USA). FITC-labeled goat anti-rabbit IgG from Abcam (Cambridge, UK), anti-P38 mAbs, anti-phosphorylated P38 (^180^Thr/^182^Tyr) mAbs and anti-caspase-3 mAb from Cell Signaling Technology (Danvers, MA, USA). Can Get Signal Immunoreaction Enhancer solutions A and B were purchased from Toyobo Co. (Osaka, Japan). 4′,6-diamidino-2-phenylindole (DAPI), RPMI 1640 medium, and fetal bovine serum (FBS) were acquired from Gibco/ Thermo Fisher (Waltham, MA, USA). Poly-L-lysine-coated slides used in this study were from MilliporeSigma (Darmstadt, Germany). Cell Counting Kit-8 (including WST-8[2-(2-methoxy-4-nitrophenyl)-3-(4-nitrophenyl)-5-(2,4-disulfophenyl)-2H-tetrazolium monosodium salt]) and HiLyte555 Labeling kit-NH_2_ were provided by Dojindo Laboratories (Kumamoto, Japan). PVDF membranes for electroblotting and peroxidase substrate EzWestBlue were obtained from ATTO Corp. (Tokyo, Japan).

### 4.2. Purification of the OXYL Lectin

OXYL was purified from stored *A. japonica* specimens as previously described [20], with minor modifications. Arms were homogenized with 10 volumes (*w*/*v*) Tris-buffer (10 mM tris(hydroxymethyl)aminomethane-HCl, pH 7.4). Homogenates were filtered through gauze and centrifuged (model Suprema 21, TOMY Co.; Tokyo Japan) at 27,500× *g* for 1 h at 4 °C. Crude supernatant was loaded in a Q-Sepharose-packed column (10 mL), which was then washed with Tris-buffer. The OXYL-containing fraction was gradually eluted with 50–300 mM NaCl in Tris-buffer, and dialyzed with Tris-buffered saline (TBS: 10 mM Tris-HCl and 150 mM NaCl, pH 7.4). The crude fraction was loaded in a fetuin-agarose column (5 mL), and OXYL was eluted with TBS containing 6 M urea. The purity of the lectin was evaluated by SDS-PAGE [73] using 15% (*w*/*v*) acrylamide gel under reducing conditions. Protein was quantified using a bicinchoninic acid protein assay kit with BSA as standard protein. Absorbance was measured at 562 nm using a 96-well microplate photoreader (SmartSpec 3000, Bio-Rad Laboratories; Hercules, CA, USA) [74,75].

### 4.3. Identification of the OXYL Coding Sequence

The N-terminal amino acid sequence of OXYL was determined with automated Edman degradation by using a protein/peptide sequencer (Shimadzu Co. Ltd., Kyoto, Japan) [5]. The partial amino acid sequence of OXYL was used as a query in a tBLASTN search [76] against two publicly available sequence resources for *A. japonica*: (i) the de novo assembled ovary transcriptome, obtained from the NCBI TSA database (mater record: GAZO00000000.1). (ii) a 30X coverage partial genome, which expected size is 650 Mbp (BioProject ID: PRJNA236227). In this case, raw sequencing data was imported in the CLC Genomics Workbench v.11 (Qiagen, Hilden, Germany), trimmed to remove sequencing adapters, low quality bases and failed reads, and de novo assembled using automatically estimated word size and bubble size parameters, and allowing a minimal contig length of 200 nucleotides. Nearly perfect matches were initially searched, by selecting hits with BLAST matches scoring e-value lower than 1 × 10^−10^. Since no significant hit could be found in the ovary transcriptome, we extended the initial BLAST hit identified in the genome to the overlapping open reading frame. The presence of possible donor and acceptors splicing sites was predicted with Genie [77], and the possible location of the poly-adenylation signal was identified by the presence of the typical eukaryotic sequence consensus AATAAA [78]. Gene structure was further confirmed by comparative means, through the multiple sequence alignment of the *A. japonica* genomic DNA with orthologous full-length cDNA sequences from other crinoids (see Section 4.4).

### 4.4. Phylogeny of OXYL

To track the evolutionary history of OXYL, all the sequences encoding a protein containing the characterizing functional domain of this lectin, the C1q domain were isolated from the available transcriptomes of crinoids and, for comparative purpose, from the fully sequenced genomes of *Strongylocentrotus purpuratus* (Echinozoa, Echinoidea), *Apostichopus japonicus* (Echinozoa. Holothuroidea) and *Acanthaster planci* (Asterozoa, Asteroidea). For Crinoidea, the following species were selected: *Antedon mediterranea*, *Cenolia trichoptera*, *Democrinus brevis*, *Dumetocrinus antarcticus*, *Florometra serratissima*, *Isometra vivipara*, *Metacrinus rotundus*, *Notocrinus virilis*, *Oligometra serripinna*, *Phrixometra nutrix*, *Promachocrinus kerguelensis*, *Psathyrometra fragilis*, and *Ptilometra australis*. All transcriptomes were de novo assembled following the protocol described in Section 4.3 and protein predictions were carried out with TransDecoder v.5.3. C1q domain containing (C1qDC) proteins were identified with hmmer v.3.1b [79], based on the detection of the C1q Pfam domain (PF00386) with a significant e-value (lower than 1 × 10^−5^). OXYL paralogous gene copies were also identified in the *A. japonica* genome assembly with a BLASTx approach (the e-value threshold was set at 1 × 10^−5^). Partial proteins with an incomplete C1q domain were discarded prior to further analysis.

All the C1qDC protein sequences identified as detailed above were used to generate a multiple sequence alignment with MUSCLE [80], which was further refined to only retain the evolutionarily informative region corresponding to the C1q domain. This dataset was preliminarily used to assess, with a Neighbor Joining clustering approach, the placement of OXYL within the phylogenetic tree of all echinoderm C1qDc proteins. A selection sequence including all the sequences putatively pertaining to a cluster of OXYL orthologous and paralogous genes was subjected to a more rigorous Bayesian inference phylogenetic analysis with MrBayes v.3.2 [81], including in the MSA the C1qDC sequences *from S. purpuratus* and the two additional C1qDC proteins detected in the *A. japonica* transcriptome as outgroups. This analysis was carried out by running two independent Monte Carlo Markov Chains in parallel for 160,000 generations, under a WAG+G+I model of molecular evolution, estimated by ModelTest-NG as the best fitting one for this dataset [82]. Run convergence was assessed by the reaching of an effective sample size higher than 200 for all the parameters of the model with Tracer (https://github.com/beast-dev/tracer/).

### 4.5. Molecular Mass Determination of OXYL

Sample concentration was estimated to be 1.0 mg/mL based on A280 measurement. Sedimentation velocity experiments were performed using an Optima XL-I analytical ultracentrifuge (Beckman Coulter; Brea, CA, USA) with An-50 Ti rotor. Standard Epon two-channel centerpiece with quartz windows were loaded with 400 μL sample and 420 μL reference solution (50 mM potassium phosphate, pH 7.4, 0.1 M NaCl). Prior to each run, the rotor was kept in a stationary state at 293 K in vacuum chamber for 1 h for temperature equilibration. A280 scans were performed without time intervals during sedimentation at 50,000 rpm, and analyzed using SEDFIT with the continuous distribution (c(s)) analysis module [83,84]. Frictional ratio (f/f_0_) was allowed to float during fitting. c(s) distribution was converted to molar mass distribution c(M).

### 4.6. Generation of Antiserum and Evaluation of Anti-OXYL Antibody Specificity

The antiserum against OXYL was raised in rabbit serum by Sigma-Aldrich. Antigen (500 μg synthesized peptide consisted of 18 amino acids of ^6^Ser-^23^Lys in OXYL) was injected 2x during 25 days, and antiserum was collected using saturated NH_3_SO_4_. Crude feather star extract and purified OXYL separated by SDS-PAGE were electroblotted on a PVDF membrane [85]. The blotted membrane was masked with TBS containing 1% (*w*/*v*) BSA, soaked with 0.2% Triton X-100 at room temperature (RT), treated with anti-OXYL rabbit serum (1:1000 dilution) (primary antibody) and HRP-conjugated goat anti-rabbit IgG (secondary antibody) for 1 h each, and colored with EzWestBlue as per the manufacturer’s instructions.

### 4.7. Immunohistochemistry of OXYL in Feather Star Tissues

Paraffin embedded sections were prepared according to the Shibata’s protocol [86]. Arms were fixed in phosphate buffered saline (PBS) containing 10% formalin overnight at RT. After washing with PBS, the specimens were decalcified in a solution containing 10% EDTA, 5% HCl, 1% formic acid and 10% sodium citrate overnight, dehydrated through a graded ethanol series, embedded in paraffin, sectioned (8 mm) and then mounted on slides. After deparaffination in xylene and hydration through the ethanol series, the sections were stained with hematoxylin-eosin. Sections were blocked with 1% (*w*/*v*) BSA containing TBS overnight at RT, treated with anti-OXYL antiserum (diluted 1:500 with PBS) and FITC-labeled anti-rabbit goat IgG for 1 h, counterstained with hematoxylin-eosin, mounted in Canada balsam, and observed by fluorescence (λ_ex/em_ = 494/520 nm for FITC) and optical microscopy. Nuclei were stained by DAPI (364/454 nm for DAPI).

### 4.8. Sugar and Glycoconjugates-Binding Specificity of OXYL

The binding specificity of OXLY to sugars and glycoconjugate derivatives was estimated using 96-well V-bottom plates. A volume of twenty μL of each solution was serially diluted in TBS, mixed with 20 μL lectin solution (previously adjusted to titer 16 [87]), trypsinized, and applied to glutaraldehyde-fixed rabbit erythrocytes in TBS containing 0.2% Triton X-100. Each plate was kept at RT for 1 h to form a dot (no agglutination; inhibited; effective) or sheet (agglutination; not inhibited; ineffective) at the bottom. For each saccharide, the minimum concentration at which each dot turned into a sheet was defined as the binding affinity to OXYL.

### 4.9. Glycan Array Analysis

Glycan array analysis was performed by Sumitomo Bakelite Co. (Tokyo, Japan). OXYL was fluorescence-labeled (λ_ex/em_ = 555/570 nm) using HiLyte Fluor 555 labeling kit-NH_2_ (Dojindo) as per the manufacturer’s instructions. A total of 8 glycans were immobilized on wells of a glycan chip (1 mM of each). Fluorescence-labeled OXYL, at concentrations ranging from 0 to 20 μg/mL, was incubated overnight at 4 °C with shielding from light. OXYL-binding glycans were detected by a Bio-REX Scan 300 evanescent fluorescence scanner (Rexxam Co. Ltd.; Osaka, Japan).

### 4.10. Bacteriostatic Assay

Bacteriostatic assays were performed as in our previous studies [12]. Gram-positive (*L. monocytogenes*) and gram-negative (*S. boydii* and *P. aeruginosa*,) bacteria were grown overnight in LB medium, harvested, and washed with phosphate-buffered saline (PBS). Fifty μL bacterial suspension (turbidity adjusted to OD_600_ = 0.6) in PBS was mixed with serial dilutions of OXYL for a quantitative assay. Bacterial suspensions were washed, and OD_600_ adjusted to 1.0. Bacteria were mixed with OXYL to final concentrations equal to 6.25, 12.5, 25, 50, 100, and 200 μg/mL in 96-well flat-type microtiter plates, and cultured at 37 °C, with OD_600_ measured every 4 h. The growth suppressive activity (%) of OXYL was calculated as (1 – (OD_600 experiment_/OD_600 control_)) × 100%.

### 4.11. Anti-Biofilm Activity of OXYL

Anti-biofilm activity was evaluated as in our previous study [12]. *P. aeruginosa* cells were grown in nutrient broth for 24 h at 30 °C. Colonies were transferred into test tubes and centrifuged at 3500× *g* for 3 min. Turbidity of bacterial cell suspensions was adjusted to OD_640_ = 1.0. Fifty μL bacterial suspension was mixed with the serial dilution of purified lectin (final volume 100 μL) in a 96-well microtiter plate, and incubated for 24 h at 37 °C. In each well, the biofilm was stained by exposure to 20 μL of 0.1% (*w*/*v*) crystal violet solution (filtered through pore size 0.45 μm filter paper) for 10 min at RT. Each well was washed 3x with TBS to remove free dye, then treated with 150 μL of 95% ethanol for 10 min at RT to release crystal violet. Extracted dye was transferred to another 96-well plate, and OD_640_ values were recorded by automated microtiter plate reader at 640 nm. The percentage of reduction of biofilm formation resulting from lectin treatment, relative to control, was calculated as follows:

% reduction of biofilm formation = (1 − (OD_640 experiment_/OD_640 control_)) × 100%

### 4.12. Binding of the Surface of Bacteria and Cultured Cancer Cells by OXYL

OXYL was labeled by HiLyte Fluor 555-labeling kit as per the manufacturer’s instructions. Cancer cell lines BT-474, MCF-7, T47D and HeLa were cultured and maintained in RPMI 1640 supplemented with heat-inactivated FBS (10%, *v*/*v*), penicillin (100 IU/mL), and streptomycin (100 μg/mL) at 37 °C in 95% air/5% CO_2_ atmosphere. Cells were washed 3× with PBS and incubated 2 h with 25 μg/mL HiLyte 555 Fluoro-labeled OXYL. Nuclei were stained by DAPI, and cells were fixed with 4% paraformaldehyde and observed by fluorescence microscopy (λ_ex/em_ = 555/570 nm for HiLyte Fluor 555; 364/454 nm for DAPI). The cytotoxic activity of OXYL (in concentrations ranging from 0–100 μg/mL) against the different cancer cell lines was determined using a Cell Counting Kit-8 containing WST-8 [16].

### 4.13. Detection of Activated Signal Transduction Molecules and their Phosphorylated Forms in BT-474 Cells in the Presence of OXYL

The human breast carcinoma cell line BT-474 (3 × 10^5^ cells) was cultured with OXYL (0–100 μg/mL) for 24 h, and cells were lysed with 200 μL cell lysis buffer M. The cell lysate was separated by SDS-PAGE and electroblotted on a PVDF membrane. The primary antibodies used in this study were directed at P38 (mouse mAb; dilution 1:3000), phospho-P38 (mouse mAb; dilution 1:3000) and caspase-3 (rabbit mAb; dilution 1:5000). Membrane was masked with TBS containing 1% BSA, soaked with 2% Triton X-100 at RT, incubated with HRP-conjugated goat anti-mouse IgG (for mouse mAb) or anti-rabbit IgG (for rabbit mAb) for 1 h [12], and colored with EzWestBlue. Experiments were performed in triplicates.

### 4.14. Statistical Analysis

For each of the studied parameters, experimental results were presented as mean ± standard error (SE) for three replicates. Data were subjected to one-way analysis of variance (ANOVA) followed by Dunnett’s test, using the SPSS Statistics software package (Chicago, IL, USA), v. 10 (www.ibm.com/products/spss-statistics). Differences with *p* < 0.05 were considered as statistically significant.

## 5. Conclusions

In this study, we characterized a type-2 LacNAc-binding 14 kDa lectin, named OXYL, from the feather star *A. japonica* (phylum Echinodermata, class Crinoidea), which proved to belong to the C1qDC family. We identified several paralogous genes in the same species, in addition to a variety of orthologs in other species of the class Crinoidea. Its tissue localization and primary structure suggest that OXYL covers a role related with immunity in this animal. Although C1qDC proteins generally create trimeric structures though a collagen tail, OXYL quaternary structure was estimated to be a multimer with tetrameric and octameric organization by analytical ultracentrifugation. OXYL caused a strong aggregation of bacterial cells, but did not affect their growth. On the other hand, the lectin displayed a LacNAc recognition-dependent anti-biofilm activity. We demonstrated that LacNAc glycans were also fundamental to enable adhesion to the surface of mammalian cultured cells. This interaction activated the MAP kinase p38, but it did not affect cell growth or affected caspase activity. The novel primary structure and the unique activities of this lectin, discovered in an organism regarded as a living fossil, highlights once again the structural and functional diversification of metazoan lectins, opening new questions about the biological role of LacNAc-binding lectin with jelly roll topology in marine invertebrates.

## Figures and Tables

**Table 1:** Structure of glycan for array analysis.

No. Name	Structures
1. Lac	Galβ1-4Glc
2. S3LN	NeuAcα2-3Galβ1-4GlcNAc
3. S6LN	NeuAcα2-6Galβ1-4GlcNAc
4. LNnT	Galβ1-3GlcNAcβ1-3Galβ1-4Glc
5. LNT	Galβ1-3GlcNAcβ1-3Galβ1-4Glc
6. NA2	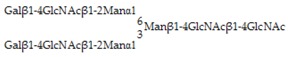
7. NA3	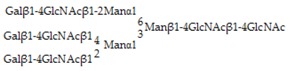
8. NA4	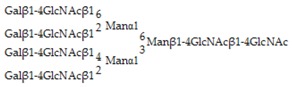

**Table 2:** Carbohydrate-binding specificity of OXYL ^1^.

**Saccharides**	Minimum inhibitory conc. (mM)
N-acetyllactosamine (Galβ1-4GlcNAc)	3.13
lactose (Galβ1-4Glc)	>100 ^2,3^
D-galactose (Gal)	>100 ^2,3^
N-acetylneuramic acid (NeuAc)	>100 ^2^
**Glycosamino glycans (GAG)**	Minimum inhibitory conc. (mg/mL)
chondroitin sulphate	>50 ^2^
heparin sodium	>50 ^2^
**Lipopolysaccharide (LPS) and Peptidoglycan (PG)**	Minimum inhibitory conc. (mg/mL)
*P. aeruginosa* lipopolysaccharide	>50 ^2^
*M. luteus* peptidoglycan	>50 ^2^

^1^ The titer of OXYL was previously diluted to 16 hemagglutination unit. ^2^ Inhibition was not observed at 100 mM (saccharides) or 50 mg/mL (GAG, LPS, and PG). ^3^ From previous results [20].

## Supplementary Materials

The following are available online at https://www.mdpi.com/1660-3397/17/2/136/s1, Figure S1: Primary structure determination of the N-terminal region of OXYL, Figure S2: Analysis of OXYL by SEDFIT program, Figure S3: Glycan-array analysis.
