# Supplementary material for: Functional Characterization of OXYL, A SghC1qDC LacNAc-specific Lectin from The Crinoid Feather Star Anneissia Japonica
Source: Mar Drugs. 2019 Feb 25;17(2):136. doi: 10.3390/md17020136 (PMC6409975; doi:10.3390/md17020136)
Supplement: Supplementary file 1 [file marinedrugs-17-00136-s001.pdf]

Supplementary Materials

Functional Characterization of OXYL, A SghC1qDC LacNAc-specific Lectin from The Crinoid Feather Star *Anneissia Japonica*

Imtiaj Hasan, Marco Gerdol, Yuki Fujii, and Yasuhiro Ozeki

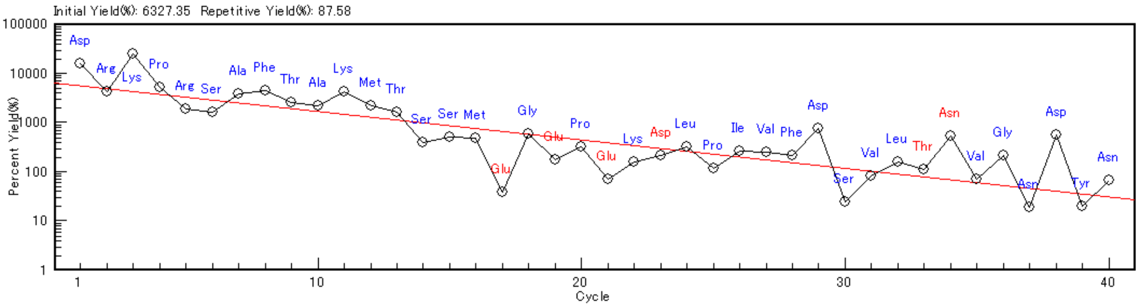

Figure S1. Primary structure determination of the N-terminal region of OXYL. Cleavage and conversion to PTH amino acid was performed by automated Edman-degradation. Four nM of OXYL were applied on the glass fiber membrane in the reaction chamber. The repetitive yield was 87.58 %.

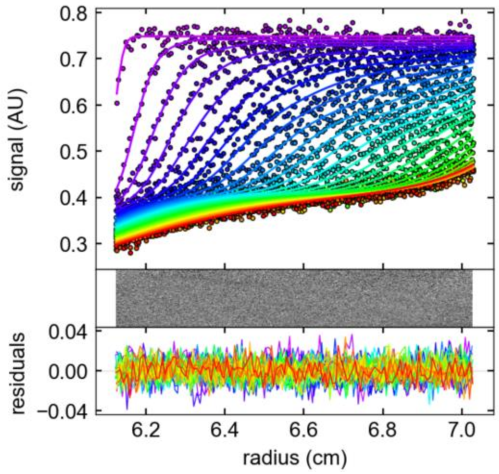

Figure S2. Sedimentation velocity data of OXYL were analyzed by SEDFIT. *Upper,* Raw data of the moving boundaries. *Lower,* Difference between the raw data and the theoretical curve.

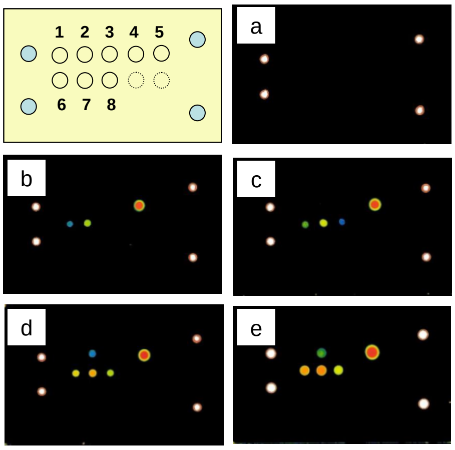

Figure S3. Glycan-array analysis. Numbers of each spot (upper left) correspond to the glycans indicated in Table 1. a to e show increasing concentrations of HiLyte555 Fluoro-labeled OXYL, i.e. 0, 1.6, 0.8, 4.0 and 20 μg/mL, respectively.
